# Molnupiravir Inhibits Replication of the Emerging SARS-CoV-2 Variants of Concern in a Hamster Infection Model

**DOI:** 10.1093/infdis/jiab361

**Published:** 2021-07-09

**Authors:** Rana Abdelnabi, Caroline S Foo, Steven De Jonghe, Piet Maes, Birgit Weynand, Johan Neyts

**Affiliations:** 1 Laboratory of Virology and Chemotherapy, Department of Microbiology, Immunology and Transplantation, Rega Institute for Medical Research, Katholieke Universiteit Leuven, Leuven, Belgium; 2 Laboratory of Clinical and Epidemiological Virology, Department of Microbiology, Immunology and Transplantation, Rega Institute, Katholieke Universiteit Leuven, Leuven, Belgium; 3 Zoonotic Infectious Diseases Unit, KU Leuven, Leuven, Belgium; 4 Department of Imaging and Pathology, Division of Translational Cell and Tissue Research, Katholieke Universiteit Leuven, Leuven, Belgium; 5 Global Virus Network, Baltimore, Maryland, USA

**Keywords:** SARS-CoV-2, antivirals, molnupiravir, VoC, hamsters, coronavirus, B.1.351

## Abstract

The emergence of SARS-CoV-2 variants of concern (VoCs) has exacerbated the COVID-19 pandemic. Currently available monoclonal antibodies and vaccines appear to have reduced efficacy against some of these VoCs. Antivirals targeting conserved proteins of SARS-CoV-2 are unlikely to be affected by mutations arising in VoCs and should therefore be effective against emerging variants. We here investigate the efficacy of molnupiravir, currently in phase 2 clinical trials, in hamsters infected with Wuhan strain or B.1.1.7 and B.1.351 variants. Molnupiravir proved to be effective against infections with each of the variants and therefore may have potential combating current and future emerging VoCs.

Since its emergence in Wuhan, China in December 2019 [1], the severe acute respiratory syndrome coronavirus 2 (SARS-CoV-2) has spread worldwide resulting in a global pandemic with more than 148 million cases and approximately 3.1 million deaths reported up to 27 April 2021 (www.covid19.who.int). Variants of SARS-CoV-2 are emerging in different parts of the world, posing a new threat of increased virus spread and potential to escape from both vaccine-induced and natural infection-induced immunity. So far, 4 major circulating SARS-CoV-2 variants of concern (VoC) have been identified: lineages B.1.1.7 (UK), B.1.351 or 501Y.V2 (South Africa), B.1.1.28.1 or P.1 (Brazil), and B.429 (California) [2]. These VoC have been implicated in new, massive waves of infections and new spikes in excess mortality in regions that have been heavily affected by SARS-CoV-2 [3]. Moreover, several vaccine candidates showed lower efficacy in phase 3 clinical trials in regions of South Africa where the VoC B.1.351 is circulating [4]. Consequently, people vaccinated against SARS-CoV-2 may not all be efficiently protected from the disease following infection with one of these new variants.

Because the emergence of new SARS-CoV-2 variants will most probably continue to happen in the future, antiviral drugs that target conserved proteins of SARS-CoV-2 could solve this issue of reduced response of variants to vaccines. Such antivirals may be expected to reduce the chance of progress to severe disease when treatment is started sufficiently early and will also have a place in a prophylactic strategy (eg, in immunodeficient patients).

The ribonucleoside analogue, *N*^4^-hydroxycytidine (EIDD-1931), was initially developed as an influenza inhibitor, but also exerts broader-spectrum antiviral activity against multiple viruses belonging to different families of RNA viruses. The molecule exerts its antiviral activity via incorporation into viral RNA, resulting in the accumulation of deleterious transition mutations in the nascent viral RNA, leading to error catastrophe [5]. Molnupiravir (EIDD-2801, MK-4482), the orally bioavailable prodrug counterpart of *N*^4^-hydroxycytidine [6], is effective against SARS-CoV-2 infections in Syrian hamsters [7], mice [8], and ferrets [9]. Data from a first-in-human, phase 1, randomized, double-blind, placebo-controlled study in healthy volunteers indicate that the drug is well tolerated and that plasma exposures exceed the expected efficacious doses based on scaling from animal models [10]. The drug is currently being assessed for its potential as an antiviral treatment of SARS-CoV-2 infection in phase 2 clinical trials of infected patients (NCT04405570, NCT04405739). Interim data from a phase 2 trial with molnupiravir demonstrated a reduction in the time required to reach negative isolation of infectious virus from nasopharyngeal swabs from participants with symptomatic SARS-CoV-2 infection [11].

We recently reported on the establishment of hamster infection models for the VoCs B1.1.7 and B.1.351. We demonstrated that no major differences in disease outcome were observed with these variants as compared to the original Wuhan strain [12]. Here, we compare the antiviral activity of molnupiravir against different SARS-CoV-2 variants in the Syrian hamster infection model.

## METHODS

**Figure 1.**
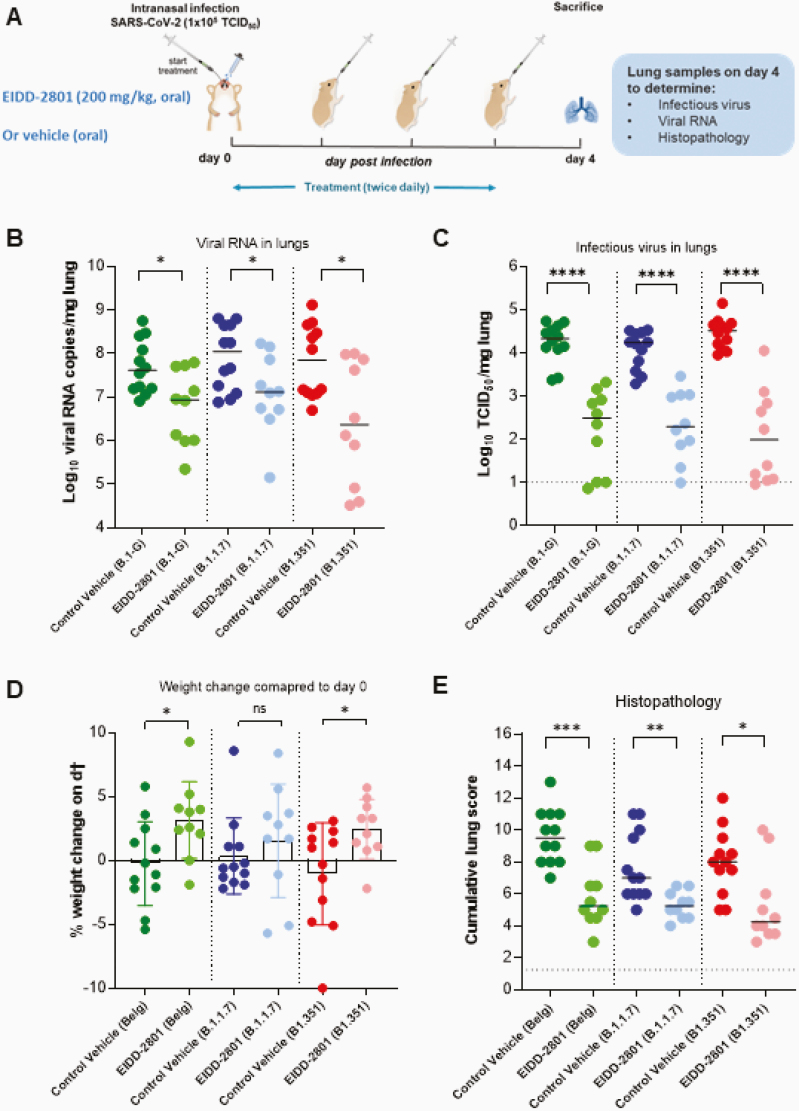
Molnupiravir (EIDD-2801) reduced viral loads in Syrian hamsters infected with different SARS-CoV-2 variants. *A*, Set-up of the study. *B*, Viral RNA levels in the lungs of control (vehicle-treated, twice a day) and EIDD-2801–treated (200 mg/kg, twice a day) hamsters infected with 10^5^ TCID_50_ B.1-G, B.1.1.7, or B.1.351 SARS-CoV-2 variants at day 4 postinfection expressed as log_10_ SARS-CoV-2 RNA genome copies per mg lung tissue. Individual data and median values are presented. *C*, Infectious viral loads in the lungs of control (vehicle-treated) and EIDD-2801–treated hamsters infected with the different SARS-CoV-2 variants at day 4 postinfection expressed as log_10_ TCID_50_ per mg lung tissue. Individual data and median values are presented. *D*, Weight change at day 4 postinfection in percentage, normalized to the body weight at the time of infection (day 0). Bars represent means ± SD. *E*, Cumulative severity score from H&E-stained slides of lungs from control (vehicle-treated) and EIDD-2801–treated SARS-CoV-2–infected hamsters. Individual data and median values are presented, and the dotted line represents the median score of untreated noninfected hamsters. All data were analyzed with the Mann-Whitney *U* test. *P < .05, **P < .01, ***P < .001, ****P < .0001. Data are from 2 independent experiments. The number of animals were 12 and 10 per vehicle and EIDD-2801–treated groups, respectively. Abbreviations: NS, not significant; SARS-CoV-2, severe acute respiratory syndrome coronavirus 2; TCID_50_, 50% tissue culture infectious dose.

All virus-related work was conducted in the high-containment biosafety level 3 facilities of the Katholieke Universiteit (KU) Leuven Rega Institute (3CAPS) under licenses AMV 30112018 SBB 219 2018 0892 and AMV 23102017 SBB 219 2017 0589 according to institutional guidelines. Briefly, 6 to 8-week-old female SG hamsters were treated orally with EIDD-2801 (200 mg/kg, twice a day) or the vehicle (ie, the control group, twice a day) for 4 consecutive days starting 1 hour before intranasal infection with 50 µL containing 1 × 10^5^ 50% tissue culture infectious dose (TCID_50_) of SARS-CoV-2 Wuhan strain (BetaCov/Belgium/GHB-03021/2020; EPI ISL 109 407976|2020-02-03) [12] or hCoV-19/Belgium/rega-12211513/2020; EPI_ISL_791333, 2020-12-21 [12] and hCoV-19/Belgium/rega-1920/2021; EPI_ISL_896474, 2021-01-11 [12], termed in brief B.1-G, B.1.1.7, and B.1.351, respectively. At day 4 postinfection, the animals were euthanized for sampling of the lungs and further analysis by intraperitoneal injection of 500 μL Dolethal (200 mg/mL sodium pentobarbital). Lungs were collected for quantification of subgenomic viral RNA using N2 primers and probes targeting the viral nucleocapsid [12], infectious virus titers, and lung histopathology, as described previously [12] (Figure 1A). Housing conditions and experimental procedures were done with the approval and under the guidelines of the ethics committee of animal experimentation of KU Leuven (license P065-2020).

## RESULTS

Molnupiravir (EIDD-2801) treatment resulted in a statistically significant reduction in the viral RNA copies per mg of lung tissue with 0.7 (*P* = .020), 0.9 (*P* = .034), and 1.5 (*P* = .016) log_10_ reduction in the groups that had been infected with B.1-G, B.1.1.7, and B.1.351, respectively (Figure 1B). Similarly, treatment significantly reduced infectious virus lung titers regardless of the SARS-CoV-2 variant used for infection (Figure 1C). The reduction in infectious virus titers in the lungs of hamsters infected with B.1-G, B.1.1.7, and B.1.351 was 1.8 (*P* < .0001), 1.9 (*P* < .0001), and 2.5 (*P* < .0001) log_10_ TCID_50_/mg tissue, respectively (Figure 1C). An increase in the percentage weight change (on day 4 compared to day 0 postinfection) was observed in the molnupiravir treated-groups compared to the corresponding vehicle-treated ones, especially following infection with the B.1-G (*P* = .020) and B.1.351 (*P* = .026) variants (Figure 1D).

**Figure 2.**
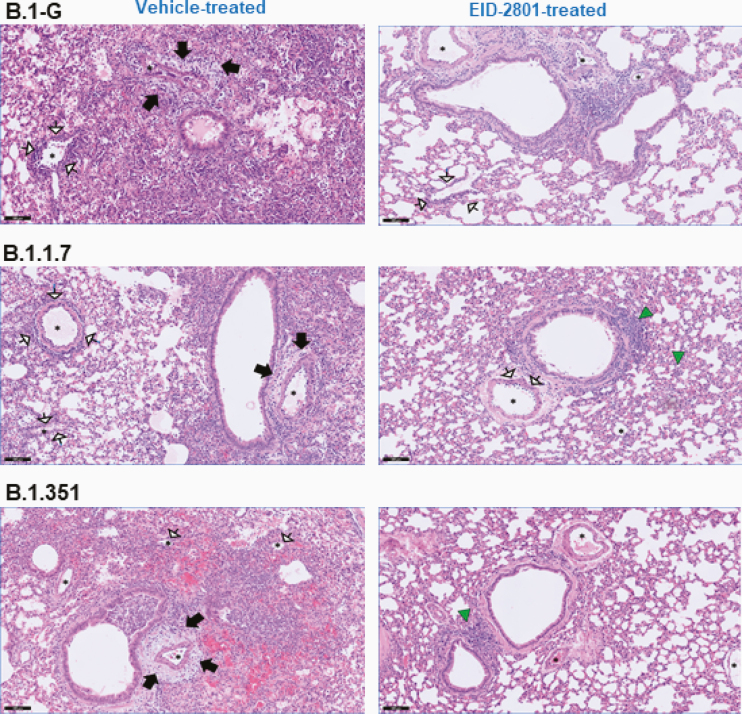
Molnupiravir improved histopathology of lungs of Syrian hamsters infected with different SARS-CoV-2 variants. Representative H&E images of lungs of control (vehicle-treated) and EIDD-2801–treated (200 mg/kg, twice a day) hamsters infected with 105 TCID_50_ B.1-G, B.1.1.7, or B.1.351 SARS-CoV-2 variants at day 4 postinfection. Left panel, the lungs of the vehicle-treated hamsters infected with B.1-G, B.1.1.7, or B.1.351 SARS-CoV-2 variants showing extensive bronchopneumonia (alveoli filled with neutrophils and histiocytes), perivascular oedema (black arrows), and perivascular cuff of inflammatory cells (arrows with white heads). Right panel, the lungs of EIDD-2801–treated groups showed no or very focal bronchopneumonia (green triangles), no or focal perivascular inflammation (arrows with white heads), and no perivascular oedema. Asterisks indicate blood vessels. Scale bar, 100 µM. Abbreviations: H&E, hematoxylin and eosin; SARS-CoV-2, severe acute respiratory syndrome coronavirus 2; TCID_50_, 50% tissue culture infectious dose.

In addition to viral loads, lung pathology was assessed using histopathological examination, as described before [12]. Significant improvement of cumulative histopathological lung scores was also observed in all the molnupiravir-treated groups with a reduction of median disease scores from 9.5 to 5.3 (*P* = .0004), 7.8 to 5.2 (*P* = .001), and 8.0 to 4.3 (*P* = .013) in molnupiravir-treated hamsters infected with B.1-G, B.1.1.7, and B.1.351, respectively, compared to the vehicle-treated controls for each variant (Figure 1E and Supplementary Table 1). Hematoxylin and eosin-stained images of lungs of the vehicle-treated hamsters infected with B.1-G, B.1.1.7, or the B.1.351 SARS-CoV-2 variants revealed extensive bronchopneumonia, perivascular oedema, and perivascular cuff of inflammatory cells (Figure 2). On the other hand, the lungs of molnupiravir (EIDD-2801)-treated animals showed no or very focal bronchopneumonia, no or focal perivascular inflammation, and no perivascular oedema (Figure 2).

## Discussion

Emerging and currently circulating VoCs present new challenges to the coronavirus disease 2019 (COVID-19) pandemic. Mutations arising in these VoCs may result in variants having altered fitness in terms of virus replication and transmission, altered interactions with key host proteins, and evasion of host immune responses [2]. With extensive mutations in the spike protein, antibody resistance of VoCs B.1.1.7 and B.1.351 have been reported [13]. B.1.351, in particular, was demonstrated to be markedly resistant to multiple monoclonal antibodies generated against the N-terminal and receptor-binding domain, as well as convalescent plasma from vaccinated individuals [13]. VoCs therefore greatly threaten the efficacies of available monoclonal antibody therapies and vaccines, which have been developed to target the parent strain of SARS-CoV-2 [4, 14].

In contrast, by acting at the level of viral RNA replication, molnupiravir should be able to exert its antiviral SARS-CoV-2 activity in spite of the mutations present in the emerging VoCs. This hypothesis is confirmed in this study, whereby molnupiravir reduces viral RNA load and infectious virus titers in the lungs of hamsters infected with parent lineage B.1-G, and VoCs B.1.1.7 and B.1.351, all to a similar extent of about 2 to 2.5 log_10_ fold compared to nontreated, infected hamsters, with comparably significant improvements in lung pathology.

The RNA-dependent RNA polymerase (RdRp) of coronaviruses is encoded by nonstructural protein 12 (nsP12), which together with the accessory proteins nsP7 and nsP8 form the core RdRp complex necessary for viral RNA replication [15]. The nsP12 consists of 3 main domains: the N-terminal nidovirus RdRp-associated nucleotidyltransferase domain, the interface domain, and the C-terminal RdRp domain [15]. The active site of the coronavirus RdRp is formed by highly conserved residues at the C-terminal domain of nsP12 [15]. Recent cryo-electron microscopy studies for the SARs-CoV-2 RdRp in presence of active metabolites of remdesivir and favipiravir revealed that both compounds were bound to the substrate-binding site of the nsP12 [15]. A proline-323-leucine substitution in the viral nsP12 is observed in B.1.1.7 and B1.351, as well as P.1 variants [2]. This amino acid residue is located in the interface domain of nsP12 and plays an important role in the interaction with the nsP8 during replication complex formation [2]. However, none of the variants carries mutations/polymorphisms in the active site of their RdRp [2]. Furthermore, given that the residues within this active site are highly conserved, nucleosides analogues such as molnupiravir are likely to remain active against new variants if they emerge.

With the efficacy of molnupiravir unaffected by mutations in VoCs B.1.1.7 and B.1.351, and taking into consideration that molnupiravir showed promising initial results in a phase 2 clinical trial in COVID-19 patients, this compound could potentially be a panlineage SARS-CoV-2 antiviral agent as more VoCs emerge in the future. Recently, we reported on the potent antiviral effect of the combination of molnupiravir and favipiravir in the SARS-CoV-2 hamster infection model [7]. By employing it as part of combination therapy, concerns for the development of resistance to molnupiravir when this drug is used alone could be greatly reduced. Consequently, molnupiravir, and other antiviral agents targeting viral replication, may be important tools in the fight against this pandemic.

## Supplementary Data

Supplementary materials are available at *The Journal of Infectious Diseases* online. Consisting of data provided by the authors to benefit the reader, the posted materials are not copyedited and are the sole responsibility of the authors, so questions or comments should be addressed to the corresponding author.

jiab361_suppl_Supplementary_Materials

## Notes

***Acknowledgments.*** We thank Carolien De Keyzer, Lindsey Bervoets, Thibault Francken, Birgit Voeten, Dagmar Buyst, Niels Cremers, Bo Corbeels, and Kathleen Van den Eynde for excellent technical assistance. We thank Professor Jef Arnout and Dr Annelies Sterckx (KU Leuven Faculty of Medicine, Biomedical Sciences Group Management) and Animalia and Biosafety Departments of KU Leuven for facilitating the animal studies.

***Author contributions.*** R. A. and J. N. designed the studies. R. A. and B. W. performed the studies and analyzed data. J. N. provided advice on the interpretation of data. R. A., C. S. F., and J. N. wrote the paper with input from coauthors. S. D. J. provided essential reagents. P. M. isolated and initially characterized variants. R. A., C. S. F., and J. N. supervised the study. J. N. acquired funding.

***Financial support.*** This work was supported by the Katholieke Universiteit Leuven/Universitair Ziekenhuis Leuven COVID-19 fund; the Fonds Wetenschappelijk Onderzoek COVID-19 call (grant number G0G4820N); the European Union Horizon 2020 Research and Innovation Program SCORE Project (grant number 101003627); and the Bill and Melinda Gates Foundation (grant number INV-00636).

***Potential conflicts of interest.*** All authors: No reported conflicts of interest. All authors have submitted the ICMJE Form for Disclosure of Potential Conflicts of Interest. Conflicts that the editors consider relevant to the content of the manuscript have been disclosed.
