# Supplementary material for: Molnupiravir Inhibits Replication of the Emerging SARS-CoV-2 Variants of Concern in a Hamster Infection Model
Source: J Infect Dis. 2021 Jul 9:jiab361. doi: 10.1093/infdis/jiab361 (PMC8408768; doi:10.1093/infdis/jiab361)
Supplement: jiab361_suppl_Supplementary_Materials [file jiab361_suppl_Supplementary_Materials.docx]

**Supplementary Table S1: Detailed histopathology scoring for hamsters infected with different SARS-CoV-2 variants and treated with either vehicle or Molnupiravir (EIDD-2801)**

| **Treatment group** | **hamster ID** | **congestion** | **intralveolar hemorrhage** | **apoptotic bodies in bronchus wall** | **necrotizing bronchiolitis** | **perivascular edema** | **bronchopneumonia** | **perivascular inflammation** | **peribronchial inflammation** | **vaculitis** | **CumulativeScore** |
| --- | --- | --- | --- | --- | --- | --- | --- | --- | --- | --- | --- |
| **Control Vehicle (B.1-G)** | 1 | 1 |  | 1 |  |  | 2 | 2 | 2 | 1 | **9** |
|  | 2 | 1 | 1 | 1 |  | 1 | 2 | 2 | 2 | 1 | **11** |
|  | 3 | 1 |  | 1 |  |  | 1 | 1 | 2 | 1 | **7** |
|  | 4 | 1 |  | 1 |  | 1 | 1 | 1 | 2 | 1 | **8** |
|  | 5 | 1 | 1 | 1 |  | 1 | 3 | 3 | 2 | 1 | **13** |
|  | 6 | 1 |  | 1 |  |  | 2 | 2 | 2 | 1 | **9** |
|  | 7 | 1 |  | 1 |  | 1 | 2 | 2 | 2 | 1 | **10** |
|  | 8 | 1 | 1 | 1 |  | 1 | 2 | 2 | 2 | 1 | **11** |
|  | 9 | 1 |  | 1 |  | 1 | 2 | 2 | 2 | 1 | **10** |
|  | 10 | 1 | 1 | 1 |  | 1 | 2 | 2 | 2 | 1 | **11** |
|  | 11 | 1 |  | 1 |  |  | 1 | 2 | 2 | 1 | **8** |
|  | 12 | 1 |  | 1 |  |  | 1 | 2 | 2 | 1 | **8** |
| **EIDD-2801 (B.1-G)**  **200 mg/kg** | 13 | 1 |  | 1 |  |  |  | 0.5 | 0.5 |  | **3** |
|  | 14 | 1 | 1 | 1 |  |  | 1 | 0.5 | 2 |  | **6.5** |
|  | 15 | 1 |  | 1 |  |  | 1 | 0.5 | 1 |  | **4.5** |
|  | 16 | 1 | 1 | 1 |  |  | 1 | 1 | 1 | 0.5 | **6.5** |
|  | 17 | 1 |  | 1 |  |  | 1 | 1 | 1 | 0.5 | **5.5** |
|  | 18 | 1 |  | 1 |  |  | 2 | 2 | 2 | 1 | **9** |
|  | 19 | 1 |  | 1 |  |  | 2 | 2 | 2 | 1 | **9** |
|  | 20 | 1 |  | 1 |  |  | 0.5 | 1 | 1 | 0.5 | **5** |
|  | 21 | 1 |  | 1 |  |  | 1 | 1 | 1 |  | **5** |
|  | 22 | 1 |  | 1 |  |  | 0.5 | 1 | 1 |  | **4.5** |
| **Control Vehicle (B.1.1.7)** | 23 | 1 |  | 1 |  |  | 0.5 | 1 | 1 | 0.5 | **5** |
|  | 24 | 1 |  | 1 |  |  | 2 | 2 | 1 |  | **7** |
|  | 25 | 1 |  | 1 |  |  | 1 | 2 | 1 |  | **6** |
|  | 26 | 1 | 1 | 1 |  |  | 3 | 3 | 1 |  | **10** |
|  | 27 | 1 |  | 1 |  |  | 2 | 2 | 1 |  | **7** |
|  | 28 | 1 |  | 1 |  |  | 1 | 2 | 1 |  | **6** |
|  | 29 | 1 | 1 | 1 |  |  | 2 | 1 | 1 | 0.5 | **7.5** |
|  | 30 | 1 |  | 1 |  |  | 3 | 3 | 2 | 1 | **11** |
|  | 31 | 1 |  | 1 |  |  | 1 | 1 | 2 |  | **6** |
|  | 32 | 1 | 1 | 1 |  |  | 1 | 1 | 1 |  | **6** |
|  | 33 | 1 | 1 | 1 |  | 1 | 3 | 2 | 1 | 1 | **11** |
|  | 34 | 1 | 1 | 1 |  |  | 2 | 2 | 2 | 0.5 | **9.5** |
| **EIDD-2801 (B.1.1.7)**  **200 mg/kg** | 35 | 1 |  | 1 |  |  | 1 | 1 | 1 | 0.5 | **5.5** |
|  | 36 | 1 |  | 1 |  |  | 2 | 2 | 0.5 |  | **6.5** |
|  | 37 | 1 |  | 1 |  |  | 1 | 1 | 1 |  | **5** |
|  | 38 | 1 |  | 1 |  |  | 1 | 1 | 1 |  | **5** |
|  | 39 | 1 |  | 1 |  |  | 1 | 0.5 | 1 |  | **4.5** |
|  | 40 | 1 |  | 1 |  |  | 1 | 0.5 | 2 |  | **5.5** |
|  | 41 | 1 |  | 1 |  |  | 1 | 0.5 | 0.5 |  | **4** |
|  | 42 | 1 | 1 | 1 |  |  | 1 | 1 | 1 |  | **6** |
|  | 43 | 1 |  | 1 |  |  | 1 | 1 | 0.5 |  | **4.5** |
|  | 44 | 1 |  |  |  |  | 3 | 2 | 0.5 |  | **6.5** |
| **Control Vehicle (B1.351)** | 45 | 1 |  | 1 |  |  | 2 | 1 | 1 |  | **6** |
|  | 46 | 1 |  | 1 |  | 1 | 3 | 2 | 0.5 |  | **8.5** |
|  | 47 | 1 |  | 1 |  |  | 1 | 1 | 1 |  | **5** |
|  | 48 | 1 |  | 1 |  |  | 1 | 1 | 1 |  | **5** |
|  | 49 | 1 |  | 1 |  |  | 2 | 2 | 2 |  | **8** |
|  | 50 | 1 | 1 | 1 |  | 1 | 3 | 3 | 1 | 1 | **12** |
|  | 51 | 1 | 1 | 1 |  | 1 | 2 | 2 | 2 | 0.5 | **10.5** |
|  | 52 | 1 |  | 1 |  |  | 2 | 2 | 2 | 0.5 | **8.5** |
|  | 53 | 1 | 1 |  |  |  | 2 | 2 | 1 | 0.5 | **7.5** |
|  | 54 | 1 |  | 1 |  |  | 1 | 1 | 1 | 0.5 | **5.5** |
|  | 55 | 1 |  | 1 |  | 1 | 3 | 2 | 1 | 0.5 | **9.5** |
|  | 56 | 1 |  | 1 |  |  | 2 | 1 | 2 | 0.5 | **7.5** |
| **EIDD-2801 (B1.351)**  **200 mg/kg** | 57 | 1 |  | 1 |  |  | 0.5 | 1 | 1 |  | **4.5** |
|  | 58 | 1 |  | 1 |  |  | 0.5 | 0.5 | 0.5 |  | **3.5** |
|  | 59 | 1 |  | 1 |  |  |  | 0.5 | 0.5 |  | **3** |
|  | 60 | 1 |  | 1 |  |  |  | 1 | 1 |  | **4** |
|  | 61 | 1 |  | 1 |  |  | 0.5 | 0.5 | 1 |  | **4** |
|  | 62 | 1 | 1 | 1 |  | 1 | 3 | 2 | 1 |  | **10** |
|  | 63 | 1 |  | 1 |  |  | 0.5 | 0.5 | 0.5 |  | **3.5** |
|  | 64 | 1 |  | 1 |  | 1 | 1 | 0.5 | 0.5 |  | **5** |
|  | 65 | 1 | 1 | 1 |  | 1 | 2 | 2 | 1 | 0.5 | **9.5** |
|  | 66 | 1 | 1 | 1 |  |  | 1 | 1 | 1 |  | **6** |
